# Signet Ring Cell Carcinoma of the Colon in Young Adults: A Case Report and Literature Review

**DOI:** 10.1155/2019/3092674

**Published:** 2019-09-11

**Authors:** Farida Abi Farraj, Hadi Sabbagh, Tarek Aridi, Najla Fakhruddin, Fadi Farhat

**Affiliations:** ^1^Faculty of Medicine, American University of Beirut, Beirut, Lebanon; ^2^Department of Pathology and Laboratory Medicine, American University of Beirut Medical Center, Beirut, Lebanon; ^3^Department of Pathology, Hammoud Hospital University Medical Center, Saida, Lebanon; ^4^Department of Oncology, Hammoud Hospital University Medical Center, Saida, Lebanon

## Abstract

Colorectal cancer (CRC), one of the leading causes of cancer-related deaths, presents with challenging features related to its diagnosis and management. The incidence of CRC in the adolescent and young adult (AYA) population has increased over the past couple of decades despite the decline in the overall occurrence of CRC in the general population. Signet ring cell carcinoma is one of the rare histopathologic subtypes of CRC; however, it is more prevalent in AYA patients than in older adults and presents with unconventional histologic characteristics, a distinct clinical behavior, and a poor prognosis. We report a case of a primary signet ring cell adenocarcinoma of the ascending colon in a 19-year-old male who presented with unusual signs and symptoms and was diagnosed with stage IVA (T4a N0 M1, with peritoneal seeding). The unusual presentation and location of the tumor in this case warrant further investigation.

## 1. Introduction

Colorectal cancer (CRC) is ranked second among women and third among men [1]. CRC overall incidence has declined over the past few years possibly due to screening by colonoscopy in patients above the age of 50 [2]. Contrarily, the incidence of CRC among patients younger than 50 years has increased at a rate of 1.5% and 1.6% in males and females, respectively. Nevertheless, CRC prevalence remains lower among young adults (20-40 years) compared to older adults (>40 years) with an incidence of 2.4% and 97.6%, respectively [3, 4].

CRC in young adults (20-40 years) has been shown to be more commonly located on the left side [4]. A recent analysis showed that younger CRC patients are more likely to present with adverse histological features, specifically vascular and perineural invasion, with positive circumferential margins after resection [5]. Also, CRC in younger patients is likely to be associated with inflammatory bowel disease, other CRC-related syndromes, and a positive family history [6]. Prognostically, current evidence is still conflicting. One population-based analysis with SEER data reported that younger patients tend to present with advanced stages but have similar outcomes as older patients [7] while others reported that younger patients tend to have poorer prognoses [8].

Signet ring cell carcinoma (SRCC) variant of CRC is an aggressive and rare entity that accounts for about less than 1% of the cases of CRC [9]. However, SRCC is more common as a CRC variant among young adults than older adults and leads to more aggressive outcomes primarily because of its late detection [9].

**Figure 1:**
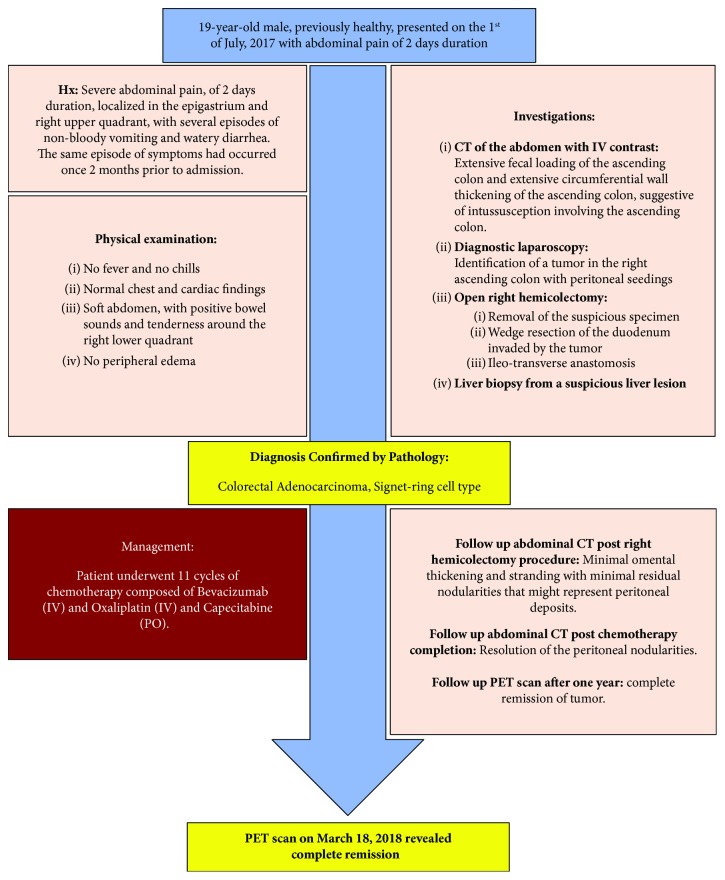
Timeline organizing the main events of the case.

This paper reports a rare case of a 19-year-old male diagnosed with stage IVA signet ring cell adenocarcinoma (T4a N0 M1, with peritoneal seeding) of the ascending colon, a location that is rather uncommon in this age group. A minireview of the literature on SRCC in younger patients is also provided. Our surgical case report (Figure 1) was written according to the Surgical CAse REports (SCARE) guidelines.

## 2. Case Presentation

**Figure 2:**
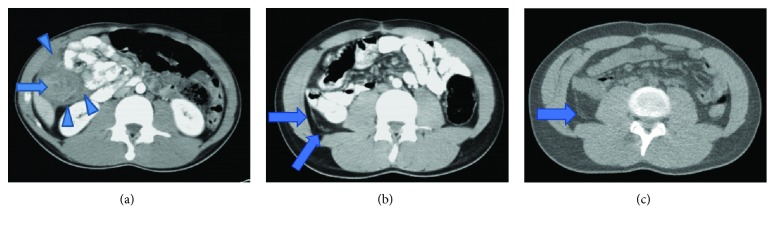
Computed tomography imaging at different stages. (a) Presurgical CT scan: infiltrative mass of the ascending colon causing significant mural thickening and luminal narrowing, associated with minimal surrounding free fluid and peritoneal nodularities. (b) Postsurgical CT scan: minimal residual nodularities at the site of surgery which can represent peritoneal deposits. (c) Postchemotherapy completion CT scan: further decrease and almost complete resolution of the peritoneal nodularities.

**Figure 3:**
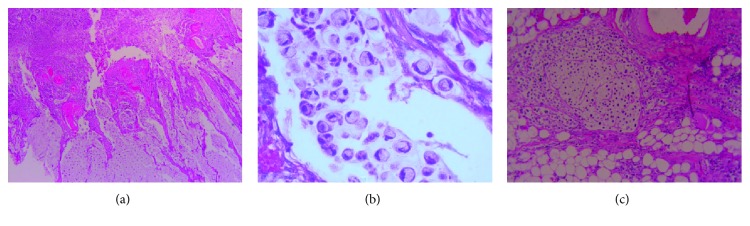
Invasive poorly differentiated carcinoma. (a) Invasive colonic signet ring carcinoma with extracellular mucin pools (200x). (b) Signet ring morphology, higher magnification (400x). (c) Signet ring tumor cells infiltrating into pericolonic fat (400x).

**Figure 4:**
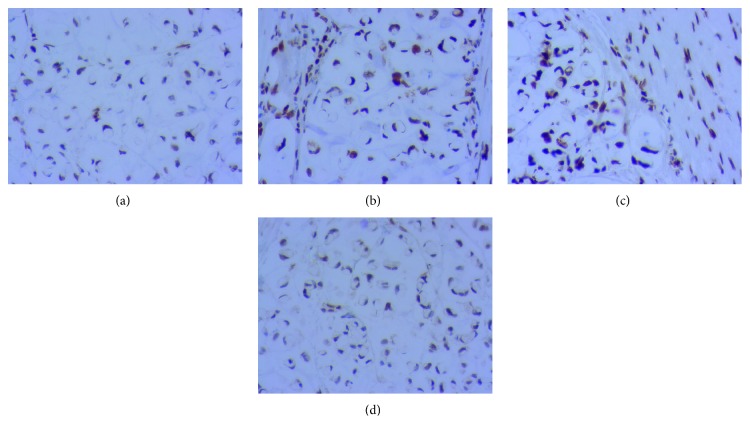
Immunohistochemistry testing for mismatch repair proteins. All proteins show intact nuclear expression: (a) MLH1, (b) MSH2, (c) MSH6, and (d) PMS2.

A previously healthy 19-year-old nonsmoker Caucasian male presented with acute epigastric pain, of 2-day duration, radiating to the right upper quadrant. Also, recurrent episodes of projectile and nonbloody vomiting as well as watery and nonbloody diarrhea occurred over a period of 2 months. He has no significant family history of gastrointestinal disease or cancer. On physical examination, the abdomen was soft with tenderness around the right lower quadrant. An abdominal CT scan revealed significant and irregular circumferential thickening of the ascending colon wall that suggests intussusception (Figure 2(a)). Subsequently, the patient underwent an exploratory laparoscopy that revealed a tumor in the ascending colon with peritoneal seeding. Right hemicolectomy followed by an ileotransverse anastomosis was performed, and a suspicious liver lesion was identified intraoperatively and resected for investigation. The pathology reported a poorly differentiated signet ring cell adenocarcinoma of the ascending colon with metastasis to the omentum and involvement of the pericolonic fat. The surgical margins were negative, with the absence of lymphovascular invasion and 24 dissected lymph nodes free of tumor. The liver biopsy was negative for metastasis. According to the TNM/AJCC staging system, the tumor is stage IV (T4a N0 M1, with peritoneal seeding) (Figure 3). Immunohistochemistry for MLH1, MSH2, MSH6, and PMS2 showed the absence of mutations for microsatellite instability (Figure 4). Gene mutation analysis by PCR sequence specific oligonucleotide probes (SSOP) for *KRAS*, *NRAS*, and *BRAF* genes detected no mutations (Table 1).

The patient was started on 11 cycles (each cycle spanned 2 weeks) of bevacizumab (400 mg) and oxaliplatin (160 mg) with 3 tablets of capecitabine (500 mg) twice daily over 10 days. The patient complied to and completed the protocol. Follow-up by MRI one month after the hemicolectomy showed an ill-defined and diffuse omental enhancement. Three months later, a CT scan of the abdomen demonstrated minimal omental thickening and stranding with minimal residual nodularities (Figure 2(b)). After completing the 11 cycles of chemotherapy, a CT scan showed resolution of the peritoneal nodularities (Figure 2(c)). Chemotherapy treatment was arrested after considering the patient's status, prognosis, and risk of recurrence. On one-year follow-up, a PET scan revealed complete remission of the tumor.

## 3. Discussion

The vast majority of signet ring cell carcinoma (SRCC) arise in the stomach. Other less common locations of this tumor include the gallbladder, pancreas, colon, rectum, bladder, and breast [10, 11]. Hence, it is important to rule out a stomach origin of tumor via imaging and biopsies. As in our case, the absence of lesions in the stomach and the presence of a colonic mass confirm the diagnosis of a primary SRCC of the colon.

SRCC is an uncommon histologic variant of CRC accounting for less than 1% of all histological subtypes. Unlike other subtypes of CRCs, SRCC among the young (ages 20-40 years) is four times more prevalent than among older adults (>40 years) [4]. SRCC is defined as a rare and aggressive malignancy of the glandular lining of the digestive tract with cells that appear as signet rings under microscopy [12]. The signet ring-like appearance of these cells results from the excess mucin accumulating within them pushing the nuclei to the peripheries. These cells are often associated with pools of extracellular mucin [13].

In this paper, we report a case of SRCC in a 19-year-old male with an uncommon presentation of intussusception by radiology and right-sided tumor location with complete remission post chemotherapy after one-year follow-up.

Classically, colorectal cancer patients present with symptoms of abdominal pain, blood in stools, unintentional weight loss, and changes in bowel habits [14]. The symptoms of colorectal SRCC are typically similar to those of colorectal cancers (Table 2). However, our case and few other cases of right-sided SRCC in young adults did not adopt the conventional presentation of CRC and rather mainly involved episodes of vomiting [15–17]. It is important to note that the latter presentation of right-sided SRCC is unique to young adults as older patients with right-sided SRCC present with classical symptoms of colorectal cancer such as abdominal pain and rectal bleed [18]. The primary workup depends on the status of the patient. A CT scan or an ultrasound imaging of the abdomen is usually part of the initial workup on a patient presenting with abdominal pain to specifically exclude an acute abdomen [19]. In our case, the patient underwent a CT scan that revealed a configuration of the colon highly suggestive of intussusception, which is a common finding in cases of colorectal cancer in young patients that might blur the diagnosis [20]. A colonoscopy and biopsy of the mucosa are also part of the workup usually following the initial imaging tests. However, because of the suggestion of intussusception, our patient was subjected to an exploratory laparoscopy that revealed a mass in the right colon and necessitated the conversion to an open laparotomy.

SRCC in younger adults is more commonly located in the left colon. Only 3 out of the 29 cases, including our case, presented with right colon involvement (Table 2). In contrast to young adults, in a retrospective review of patients with SRCC, with most cases being above 40 years of age, SRCC in older adults was shown to be more prevalent in the right colon [18]. Right colon tumors are known to have a worse prognosis with lower 5-year specific survival rate and 5-year disease-free survival rate [20]. The worse prognosis could be attributed to the different genetic and epigenetic alterations between right and left colorectal cancer, as well as the different molecular pathways that affect the course of the disease and impact its management [21]. Patients with signet ring cell carcinoma of the colon tend to have an overall poor survival rate compared to patients with other histologic subtypes, mainly due to the vague and late presentation of the symptoms [21]. A recent multivariate adjusted survival analysis on the National Cancer Data Base (NCDB) showed that signet ring cell histology is associated with 57% higher risk of death relative to nonmucinous and nonsignet ring adenocarcinoma for both colon and rectal cancers [9]. Another surveillance study compared the survival of patients with right-sided SRCC to patients with left-sided SRCC and revealed that patients with right-sided SRCC had lower mortality rates in stage II disease [22]. Such an association in younger adults with SRCC is to be established yet.

Recent studies proved the effective role of adjunct chemotherapy, in accord with surgical resection of the tumor, in late stages of SRCC and prolonging the overall survival [23]. In our case, the therapeutic protocol consisted of 3 chemotherapeutic regimens following the surgical resection of the tumor and proved to be successful in treating the patient.

The molecular profile of SRCC is associated with lower prevalence of *KRAS* and *NRAS* mutations, higher prevalence of *BRAF* and CIMP (CpG island methylator phenotype) mutations, and a lower expression of p16 and p53 [24]. However, SRCC has been associated with a significantly reduced expression of E-cadherin adhesion protein that complexes with catenin proteins to maintain the polarity of epithelial cells. The invasive phenotype of SRCC is enhanced by the acquired motility of cells owing to the reduced expression of E-cadherin molecules [25]. *KRAS*, *NRAS*, and *BRAF* gene mutations were not detected in this case (Table 1). A recent whole-exome and RNA sequencing study demonstrated that the difference concerning the molecular basis of colorectal SRCC and nonsignet ring conventional colorectal adenocarcinoma is that the former is usually richer in SRCC-specific genes that result in epithelial-mesenchymal transition (EMT) and stem cell upregulation which contribute to the invasiveness of such tumors [26].

SRCC is associated with distant lymph node metastasis, in addition to more advanced stages at presentation, which significantly worsen its prognosis relative to other colorectal cancers [9, 24]. The literature on young SRCC patients revealed 17 out of 29 cases with lymph node involvement and 14 out of 29 cases with distant metastasis (Table 2). Our case presented with metastasis to the omentum and involvement of the pericolonic fat, with the absence of metastasis to the lymph nodes and liver.

Studies on young patients (<40 years) that have SRCC of colorectal origin are scarce; about 29 cases have only been reported in the literature (Table 2). A history of inflammatory bowel disease like ulcerative colitis (2/29) and Crohn's disease (1/29) is not common in young SRCC patients. This finding was similar in older adult SRCC patients [18]. A positive family history was reported in 4 out of 29 cases only; and 3 out of 29 cases had polyps. Metastasis was reported in around half of the cases (14/29). Additionally, 18 out of the 29 patients received chemotherapy with only 5 long-term survivors. The rest of the patients with distant metastasis had either died (5/14) or were lost to follow-up (5/14). Among the 12 patients with localized disease, only one of them died. These findings stress the importance of early diagnosis and treatment.

## 4. Conclusion

We conclude that SRCC, a rare histopathologic subtype of CRC that is more prevalent in young adults than in older adults, has a distinct clinical presentation and an unconventional localization in the colon that warrant a conclusive workup. It is important to consider SRCC in our differential of an AYA patient presenting with prolonged and vague gastrointestinal symptoms, as SRCC is an aggressive subtype of CRC with poorer prognosis if detected in an advanced state. Moreover, it is important to rule out a primary GI malignancy that has metastasized to an atypical location in the colon. Additional assessment of the molecular characteristics of this aggressive subtype of CRC further improves the therapeutic modalities of this disease that can result in a good prognosis.

## Figures and Tables

**Table 1:** The mutations tested and their corresponding genes for our patient.

Gene	Mutations
*KRAS*	Codons: 12-13-59-60-61-117-146
*NRAS*	Codons:12-13-59-60-61-146
*BRAF*	V600A-V600D-V600E-V600G-V600K-V600M-V600R-K601E

**Table 2:** Main clinicopathological parameters of SRCC of the colon cases reported in the literature.

No.	Citations	Age (years)	Gender	History of IBD	Family history	Polyps	Location	Tumor size (cm)	Lymph nodes	Distant metastasis	Chemotherapy	Survival
1	Seo et al. [27]	39	M	UC	N/A	−	R	0.8	+	−	N/A	Alive
2	Tamai et al. [28]	37	F	N/A	N/A	−	TC	7.0	N/A	+	−	Deceased
3	Shimizu et al. [29]	38	M	UC	N/A	+	R	1.5	−	−	N/A	N/A
4	Posey et al. [30]	25	M	N/A	N/A	−	R	N/A	+	+	+	Deceased
5	Nakata et al. [31]	22	F	N/A	−	−	DC	1.5	−	−	N/A	Alive
6	Achneck et al. [32]	30	F	−	N/A	−	R	10.6	+	−	N/A	Alive
7	Kilickap et al. [33]	29	M	N/A	N/A	−	R	N/A	+	+	+	N/A
8	Maltz and Schwartz [34]	35	M	CD	N/A	−	SC	N/A	+	+	N/A	Deceased
9	Derici et al. [35]	23	M	−	+	−	R	N/A	+	−	−	Deceased
10	Selcukbiricik et al. [36]	37	F	N/A	N/A	−	SC	N/A	+	+	+	N/A
11	Canepa et al. [37]	40	M	−	N/A	−	R	7.0	+	−	+	N/A
12	Charles et al. [38]	24	M	N/A	+	−	R	N/A	+	+	+	N/A
13	Kang et al. [10]	21	M	−	N/A	+	R	0.5	−	−	N/A	N/A
14	Mehta et al. [39]	37	M	N/A	+	−	SC	N/A	+	+	+	N/A
15	Pamukçu et al. [19]	19	M	N/A	N/A	−	SC	N/A	N/A	N/A	+	N/A
16	Richer et al. [15]	19	F	N/A	+	−	AC	6.0	+	N/A	N/A	N/A
17	Shaaban et al. [40]	30	F	N/A	N/A	−	R	6.0	+	+	+	Deceased
18	Kendre et al. [41]	34	M	N/A	N/A	−	R	N/A	+	+	+	Alive
19	Prabhu et al. [16]	28	F	−	N/A	−	TC	N/A	−	−	+	Alive
20	Dhull et al. [42]	26	F	N/A	N/A	−	R	10.0	−	−	+	N/A
21	Santos-Antunes et al. [43]	32	M	N/A	N/A	−	AC	N/A	N/A	N/A	−	Deceased
22	Yadav et al. [44]	19	M	N/A	N/A	−	RC	N/A	+	−	+	Alive
23	Park et al. [45]	36	F	N/A	−	−	C	4.9	−	−	+	N/A
24	Turati et al. [46]	29	M	N/A	N/A	−	R	8.3	+	+	+	Alive
25	Zhou et al. [47]	27	M	N/A	N/A	−	TC	N/A	N/A	+	N/A	Alive
26	Khan et al. [17]	20	M	N/A	N/A	+	TC	N/A	+	+	+	N/A
27	Lusilla et al. [48]	25	M	N/A	N/A	−	R	N/A	−	+	+	Deceased
28	Ren et al. [49]	31	F	−	N/A	−	R	N/A	+	−	+	N/A
29	Our case	19	M	−	−	−	AC	N/A	−	+	+	Alive

M: male; F: female; IBD: inflammatory bowel disease; CD: Crohn's disease; UC: ulcerative colitis; R: rectum; TC: transverse colon; DC: descending colon; SC: sigmoid colon; AC: ascending colon; C: cecum; N/A: not available.

## Abbreviations

CD: Crohn's disease
CRC: Colorectal cancer
CT: Computed tomography
IBD: Inflammatory bowel disease
MRI: Magnetic resonance imaging
NCDB: National Cancer Data Base
PCR: Polymerase chain reaction
PET: Positron emission tomography
SRCC: Signet ring cell carcinoma
SCARE: Surgical CAse REports
UC: Ulcerative colitis.
